# Noninvasive Monitoring of Palmitoyl Hexapeptide-12 in Human Skin Layers: Mechanical Interaction with Skin Components and Its Potential Skincare Benefits

**DOI:** 10.1021/acsabm.4c01816

**Published:** 2025-02-18

**Authors:** Cosimo Ligorio, Elham Tavasoli, Nevena Karaman-Jurukovska, Abraham Ittycheri, Anna M. Kotowska, Mohammed H. Khan, David J. Scurr, Shovit A. Gupta, Leah V. Moogan, Jaime Emmetsberger, Fake Lu, Guy K. German, Tom Mammone, Alvaro Mata

**Affiliations:** † Biodiscovery Institute, University of Nottingham, Nottingham NG7 2RD, United Kingdom; ‡ Department of Chemical and Environmental Engineering, University of Nottingham, Nottingham NG7 2RD, United Kingdom; § School of Pharmacy, University of Nottingham, Nottingham NG7 2RD, United Kingdom; ∥ Advanced Technology Pioneering, The Estée Lauder Companies, Melville, New York 11747-3115, United States; ⊥ Department of Biomedical Engineering, Binghamton University, State University of New York, Binghamton, New York 13902-4400, United States; # Materials Science and Engineering, Binghamton University, State University of New York, Binghamton, New York 13902-4400, United States; ∇ Department of Pharmaceutical Sciences, Binghamton University, State University of New York, Binghamton, New York 13902-4400, United States

**Keywords:** Palmitoyl hexapeptide-12, self-assembling peptides, cosmetics, OrbiSIMS, SRS, human skin

## Abstract

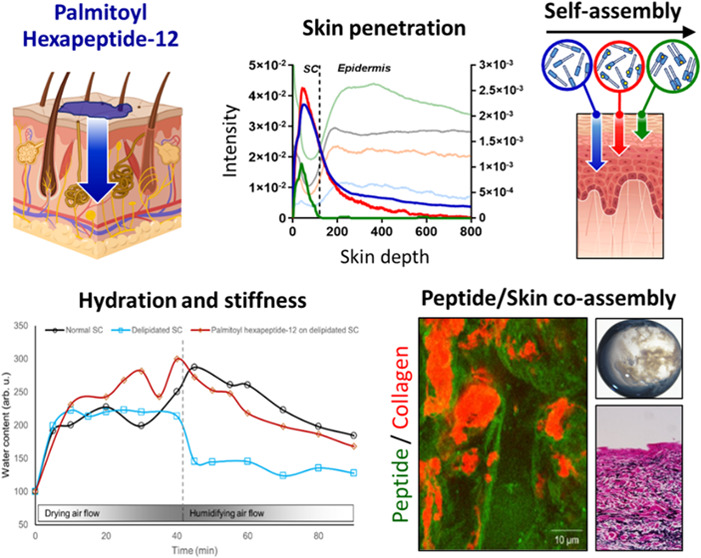

Self-assembling peptides (SAPs) represent a rich source of building blocks that interact with biological structures. For instance, cosmetic SAPs like Palmitoyl hexapeptide-12 have gained increasing interest for their anti-aging properties. However, their short-term impact on the skin composition and mechanics remains unclear. In this study, a battery of label-free techniques is exploited to objectively monitor the effects of Palmitoyl hexapeptide-12 on human skin. Orbital trapping secondary ion mass spectrometry (OrbiSIMS) is used to discern between Palmitoyl hexapeptide-12 sol and gel forms, tracking its self-assembly and penetration within full-thickness human skin. Palmitoyl hexapeptide-12 is shown to permeate both stratum corneum and epidermal layers, initiating gel formation by harnessing endogenous ions. Hence, the ability of the peptide to strengthen and repair the skin barrier after delipidation is also demonstrated through a high-throughput mechanical characterization and stimulated Raman scattering (SRS). Finally, the co-assembling properties of Palmitoyl hexapeptide-12 with native skin molecules are shown via *in vitro* tests and *ex vivo* histology. This study establishes a methodological benchmark for measuring the effects of cosmetic peptides on skin mechanics and hydration, introducing a platform to design SAPs capable of harnessing native skin molecules to create “biocooperative” structures with cosmetic benefits.

## 1 Introduction

Self-assembly enables the spontaneous interaction and arrangement of individual molecules into well-defined, higher-order structures. Consequently, there is increasing interest in exploiting self-assembly as a tool to develop synthetic materials that can emulate or interact with biological systems. Self-assembling peptides (SAPs), such as RADA-16 and FEFKFEFK, have been largely used as three-dimensional (3D) models for cell culture of primary and stem cells,^1,2^ as well as hemostats for accelerated wound healing.^3^ Similarly, SAPs based on β-hairpins have been employed as injectable cell carriers for minimally invasive applications,^4^ while Fmoc-capped aromatic peptides opened the way for stimuli-responsive adaptive systems.^5^ In the field of regenerative medicine, peptide amphiphiles (PAs), pioneered by Stupp and colleagues, have the capacity to assemble into nanofibrous matrices displaying a wide range of bioactive functionalities,^6^ shown to promote tissue regeneration,^7^ sequester growth factors,^8^ or serve as templates for mineralization.^9^ Overall, peptides represent a rich resource of building blocks to engineer materials that can interact with biological structures.

Recently, the field of skincare has witnessed an increasing interest in the use of peptides.^10^ As cosmetic ingredients, peptides can mimic the structure and function of extracellular matrix (ECM) components such as collagen and elastin, which constitute the molecular framework of the skin. Therefore, it is not surprising that many peptides used for skincare, also known as matrikines, are derived from ECM components, such as collagen and elastin.^11^ Compared to other peptide-based skin treatments, matrikines can interact directly with protein epitopes and cell receptors to stimulate ECM synthesis, induce repair, and act as mediators of tissue remodeling.^11^ Matrikines can show unique broad-range and signaling properties in the skin, as they are known to act synergistically *in vivo* to produce greater tissue repair and antiaging effects.^12^ Moreover, in the case of palmitoylated peptides, greater skin permeation has been shown compared to unmodified conventional peptides.^13^ For instance, the Palmitoyl pentapeptide-4 (pal-KTTKS), derived from collagen type αI, has shown *in vitro* elevated expression of fibronectin, hyaluronic acid (HA), and collagen type I and III,^14^ while as a topical agent, it has shown to reduce wrinkles and age spots, and restored skin elasticity.^15^ Similarly, Palmitoyl hexapeptide-12 (pal-VGVAPG), derived from elastin,^16^ has been shown to be a chemoattractant for skin fibroblasts *in vitro*(17) and successfully improved skin elasticity and tone when applied topically *in vivo*.^18^ Both peptides have been shown to cross the skin barrier while acting as moisturizers.^19^ Given their versatility and functionality, peptides offer exciting opportunities in skincare applications and cosmetics.

Currently evaluation of performance is based primarily on visual examination of skin, rather than examining and quantifying molecular composition and tissue structure.^20^ Proper demonstration of peptide efficacy should be accompanied by rigorous and quantifiable data whenever possible while considering important ethical testing considerations. Another important limitation consists of the need for peptide-based materials to diffuse through the skin and assemble into stable and robust structures within the skin. Indeed, peptides are highly susceptible to protease enzymes in the skin, which can cause peptide breakdown.^21^ Overcoming these challenges would further open opportunities for the use of SAPs as topical products in skincare or cosmetic applications.

SAPs can be used as part of structural multicomponent self-assembling materials.^22^ For example, PAs have been shown to co-assemble into robust nanofibrous hydrogels when combined with recombinant proteins such as elastin-like^23^ or resilin-like^24^ polypeptides as well as with polysaccharides such as HA^25^ or xyloglucan.^26^ This approach can be exploited to co-assemble PAs with multiple ECM components such as collagen, fibronectin, and HA^27^ or even more complex fluids such as artificial sputum medium,^28^ blood,^29^ and amniotic fluid.^30^ This co-assembling approach can also be exploited with different kinds of SAPs, such as multi-domain^31^ or short aromatic peptides^32^ co-assembling with HA or ELPs, respectively. These examples demonstrate the possibility of using multiple ECM components present in skin, both as triggers of SAP assembly and as building blocks with which SAPs can be co-assembled. This feature could be exploited to co-assemble cosmetic peptides with native molecules and macromolecules present in the skin to create “biocooperative” supramolecular assemblies, able to provide perceivable skin benefits including tissue hydration and enhanced skin elasticity.

## 2 Results and Discussion

### 2.1 Rationale of the Study

Palmitoyl hexapeptide-12 is currently utilized in topical skincare products for its proven anti-aging benefits by triggering various biological signaling pathways when they permeate the skin. Our hypothesis is that this peptide may mechanically interact with the major compositional skin components to offer benefits without involving biogenesis. We propose that this peptide may integrate with lipid components when residing in more superficial skin layers, such as the stratum corneum, altering the drying kinetics of the skin, and thereby enhancing water retention and skin barrier strengthening. Additionally, we demonstrate that the peptide can self-assemble with ions, such as Ca^2+^, and co-assemble with native macromolecules found in the skin’s dermal layer, such as hyaluronic acid (HA), collagen type I, and elastin, forming nanofibrous hydrogels while penetrating deeper into the dermis. Hence, these phenomena may have the potential to mechanically impact skin properties in shorter periods of time without involving biogenesis, resulting in the faster achievement of skin benefits.

The permeation of human skin by Palmitoyl hexapeptide-12 was first investigated using orbital trapping secondary ion mass spectrometry (OrbiSIMS), to perform a label-free *in situ* analysis of peptide permeation. This allowed non-destructive analysis of the peptide’s depth and spatial distribution within human skin layers. Moreover, due to the exceptional mass accuracy of OrbiSIMS, this technique has been used also to discriminate between the cosmetic peptide in its solution and gel forms. This information guided us to identify the supramolecular assembly of the cosmetic peptide while it is permeating within full-thickness human skin samples. Subsequently, we used Stimulated Raman Scattering (SRS) microscopy as a chemical-specific imaging tool to map the distribution of peptides as they penetrated the stratum corneum (SC) and to evaluate their impact on skin mechanics and hydration after treatment. We also characterized the barrier function of the human SC using a high-throughput mechanical characterization method to demonstrate the advantages of this peptide in strengthening and repairing the skin barrier.

Following this, *in vitro* experiments were conducted to demonstrate the interaction of Palmitoyl hexapeptide-12 with major ECM components of the dermis. In particular, collagen type I, elastin, and hyaluronic acid (HA) were studied as the main components responsible for the structural integrity of the skin. Finally, histological staining was used to detect the effect of Palmitoyl hexapeptide-12 on the elastin fibers in decellularized *ex vivo* human skin.

### 2.2 Peptide Penetration and Assembly in an *Ex Vivo* Human Skin Model

#### 2.2.1 Discerning between Non-assembled and Self-Assembled Peptides

**Figure 1:**
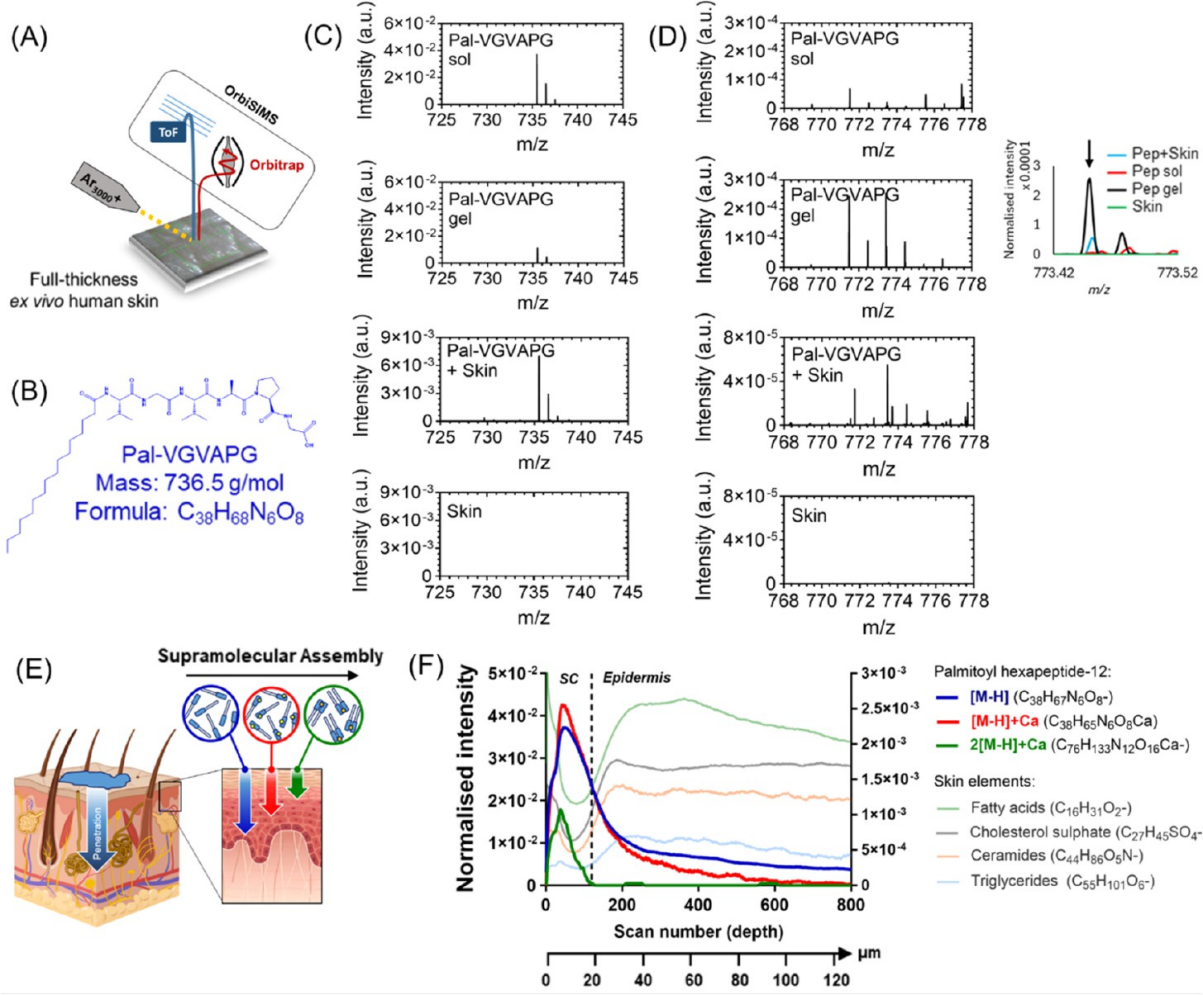
OrbiSIMS analysis of peptide penetration in human skin. (A) Schematics of the OrbiSIMS experiment conducted on *ex vivo* human skin. (B) Chemical formula and molecular mass of Palmitoyl hexapeptide-12. (C) Negative polarity spectra of Palmitoyl hexapeptide-12 in its solution (“sol”) and hydrogel (“gel”) forms show a unique peak for the sol peptide at *m*/*z* 735.5 ([M–H], formula: C_38_H_67_N_6_O_8_^–^) and (D) a unique peak for the gel peptide at *m*/*z* 771.4785 g/mol ([M–H]+Ca, formula: C_38_H_65_N_6_O_8_Ca^–^). Inset on the right shows the secondary ion at *m*/*z* 773.4514 present in the gel but absent in the sol form. (E) Schematics of the peptide penetration and hierarchical assembly within the human skin. (F) OrbiSIMS negative polarity depth profile of Palmitoyl hexapeptide-12 in its monomeric form (blue depth profile and circle), as monomers interacting with calcium atoms (red depth profile and circle), and as dimers interacting with calcium atoms (green depth profile and circle). Intensity of the red spectrum is referred to the right *Y*-axis. Green depth profile has been multiplied by a factor 100. In the background, spectra of skin elements, such as fatty acids, cholesterol sulfate, ceramides, triglycerides, and amino acids are displayed. A dashed vertical line indicates the physical transition from the stratum corneum (SC) and the underlying epidermis.

As an initial screening step, we used OrbiSIMS to investigate the permeation, spatial distribution, and penetration depth of Palmitoyl hexapeptide-12 through the layers of an *ex vivo* human skin model (Figure 1A). The method applied here to depth profile skin from the outermost layer has been previously successfully applied to observe native chemistry as well as trace the permeation of applied exogenous active compounds..^33^ Here, the self-assembling behavior of Palmitoyl hexapeptide-12 peptide in human skin was also investigated, by analyzing the peptide in its non-assembled (“sol”) and self-assembled (“gel”) form. This investigation on one side would confirm the capability of Palmitoyl hexapeptide-12 of penetrating across layers of human skin; on the other side it would assess the possibility for the peptide to assemble within human skin and provide its location in the tissue. To achieve this, we initially examined OrbiSIMS spectra to identify molecular peaks unique to Palmitoyl hexapeptide-12 in its sol and gel form. Based on the literature on amphiphilic SAPs, we understand that peptide monomers will start to self-assemble into supramolecular structures by hydrophobic interactions occurring between the tails of two or more monomeric building blocks.^34^ As shown in Figure 1B, the molecular mass of Palmitoyl hexapeptide-12 peptide monomers is 736.5 g/mol with a molecular formula of C_38_H_68_N_6_O_8_. Being Palmitoyl hexapeptide-12 monomers are the building blocks of its supramolecular assemblies, it is expected that Palmitoyl hexapeptide-12 in its sol and gel forms would share a common secondary ion at *m*/*z* 735.50, which corresponds to the deprotonated mass of the peptide monomer ([M–H], formula: C_38_H_67_N_6_O_8_^–^). This peak was absent in the spectrum of the native untreated skin, while it was present in the spectrum of the human skin treated with 1% Palmitoyl hexapeptide-12 solution (Figure 1C). We also confirmed that the secondary ion at *m*/*z* 735.50 was unique to Palmitoyl hexapeptide-12 and did not come from other lipid structures present in the skin as it was absent in the lipid mass spectral database.^35^ Following this initial check, our second step aimed to find a unique peak for the Palmitoyl hexapeptide-12 peptides in their assembled state. We observed that gel samples of Palmitoyl hexapeptide-12 possessed a unique peak at *m*/*z* 773.4514 (C_38_H_65_N_6_O_8_Ca^–^), which was also present in human skin treated with the peptide solutions (Figure 1D). Conversely, this secondary ion was absent for 1% Palmitoyl hexapeptide-12 sol, and it was absent in the untreated human skin (Figure 1D, inset). The identified peak corresponded to the molecular mass of the peptide monomer plus one calcium atom ([M–H]+Ca, formula: C_38_H_65_N_6_O_8_Ca^–^), which suggested that Palmitoyl hexapeptide-12 monomers start to exploit ionic compounds present in the skin as gelation triggers. In particular, gradients of calcium ion signaling are known to be present in the human skin, both in SC and epidermis,^36^ where calcium regulates keratinocytes differentiation^37^ and permeability of the epidermal barrier.^38^ These results showed that OrbiSIMS represents a label-free, powerful technique to discern between peptides in their nonassembled (“sol”) and self-assembled (“gel”) forms within skin tissue.

#### 2.2.2 Depth Profile of Peptide Penetration within Human Skin

Having identified the unique OrbiSIMS molecular secondary ions for Palmitoyl hexapeptide-12 as a gel and solution, we used them to evaluate the peptide penetration and self-assembly within an *ex vivo* human skin model (Figure 1E). As reported previously, the human skin is composed by multiple molecular species, including fatty acids and cholesterol esters (formula: C*_n_*H*_n_*O_2_), sulfate-based lipids (C*_n_*H*_n_*S_1_O*_n_*), triglycerides (C*_n_*H*_n_*O_6_), ceramides, amino acids and their derivatives (C*_n_*H*_n_*O*_n_*N_1_).^33^ In particular, it has been shown that these different compounds exhibit different trends with depth away from the surface of the skin, allowing OrbiSIMS to distinguish between the molecular compositions and locations of skin layers, such as the SC and the underlying epidermis.^33^ From the OrbiSIMS analysis of treated skin, it is evident that Palmitoyl hexapeptide-12 in its monomeric form penetrated through the SC and beyond (blue line depth profile) with almost a constant intensity throughout the epidermal layer (Figure 1F). We hypothesize that during penetration, Palmitoyl hexapeptide-12 monomers interact with calcium atoms present in the skin and self-assemble (red line depth profile), represented by a secondary ion corresponding to *m*/*z* 773.4514 (formula: C_38_H_65_N_6_O_8_Ca^–^) that was observed in the SC and underlying epidermis (Figure 1F). Exhibiting a similar depth distribution at a reduced ion intensity, we also observed a molecule with a mass of *m*/*z* 1509.96 (green line depth profile), which represents the supramolecular assembly of two Palmitoyl hexapeptide-12 monomers plus a calcium atom (2[M–H]+Ca, formula: C_76_H_133_N_12_O_16_Ca^–^) (Figure 1F). We confirmed that these peaks were uniquely representative of Palmitoyl hexapeptide-12 assemblies as they were not present in the lipid mass spectroscopy database. This ion is also unique to the gel form of Palmitoyl hexapeptide-12 and is not present in the spectra of Palmitoyl hexapeptide-12 as sol. We speculate that calcium ions present in the skin create ionic bridges between multiple peptide monomers, whose carboxylic groups are deprotonated at physiological pH. Taken together, these results show topical peptides starting to harness and co-assemble with elements present in the native skin, particularly with calcium atoms. In terms of depth penetration, considering the stratum corneum being approximately 20 μm in depth and assuming a uniform depth profiling rate throughout the tissue, we estimate that Palmitoyl hexapeptide-12 monomers ([M–H]) penetrated >∼100 μm into the epidermis layer thickness. Conversely, Palmitoyl hexapeptide-12 supramolecular assemblies with ions, such as [M–H]+Ca, were located in the epidermis, up to 80 μm below the SC. The calcium-mediated assembly of peptides within the skin could offer remarkable advantages for advanced skincare and therapeutic delivery. First, the gelation of Palmitoyl hexapeptide-12 within the skin could protect its molecular structure from premature enzymatic degradation, a common disadvantage for cosmetic formulations,^39^ enhancing peptide stability and bioavailability. Second, the bioresponsive behavior of SAPs allows for adaptive interactions and dynamic function within the skin microenvironment. For instance, the mammalian epidermis exhibits a distinctive calcium gradient between its lower and upper layers.^38^ This natural feature could be leveraged to achieve a tailored peptide assembly within specific zones of the skin, enabling precise targeting and localized therapeutic effects. Finally, harnessing naturally occurring calcium ions in the skin for assembly may lead to improved biocompatibility and reduced risk of adverse reactions, compared to other toxic cross-linkers.^40^ Here, we demonstrated the feasibility and potential of using OrbiSIMS to observe the penetration and supramolecular assembly of peptides throughout multiple layers of human skin.

#### 2.2.3 Spatial Peptide Distribution on Human Skin

**Figure 2:**
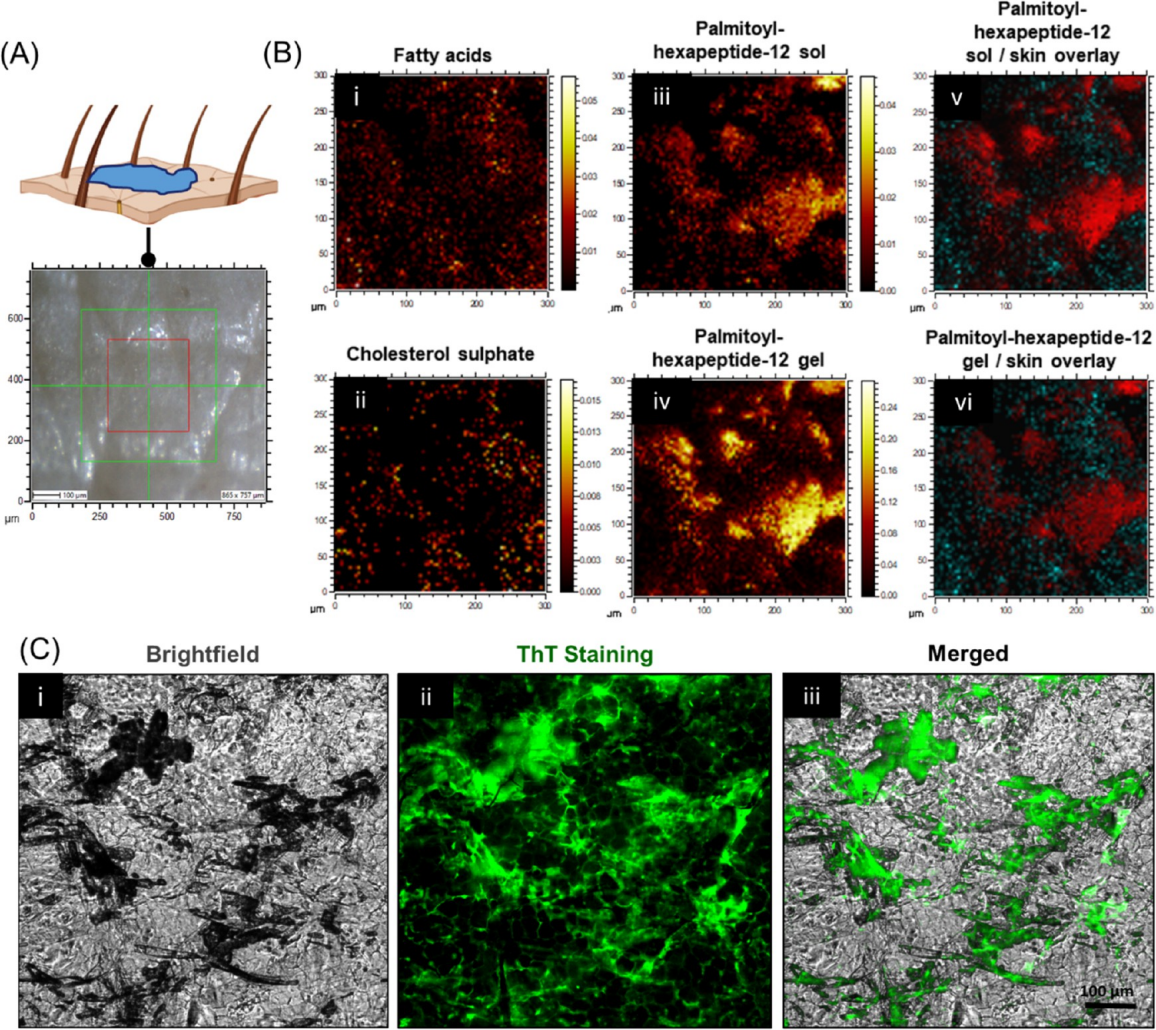
(A) Peptide application and image of the area of detection. (B) OrbiSIMS high-resolution negative polarity ion images of fatty acids and cholesterol sulfate (i and ii), Palmitoyl hexapeptide-12 in sol and gel forms (iii and iv), and overlay of the cosmetic peptide with fatty acids (v) and cholesterol sulfate (vi). (C) Images of the cosmetic peptide on the surface of human skin in brightfield mode (i), stained with Thioflavin T (ThT) dye (ii), and merged composite image (iii). ThT staining shows the spatial distribution of the assembled Palmitoyl hexapeptide-12 fibers.

Alongside a depth profile investigation, we undertook a secondary ion image analysis to demonstrate the capability of OrbiSIMS to image the spatial distribution of Palmitoyl hexapeptide-12 peptides on the surface of human skin following Franz cell diffusion (Figure 2A). Secondary ion images were retrospectively constructed for fatty acids and cholesterol sulfate as skin references, while we looked at Palmitoyl hexapeptide-12 monomers (sol form, *m*/*z* = 735.5) and Palmitoyl hexapeptide-12 assembled with calcium atoms (gel form, *m*/*z* = 773.4514) as peptide samples. Palmitoyl hexapeptide-12 monomers were distributed in the form of high-intensity ion patches on the surface of the skin (Figure 2B). Interestingly, Palmitoyl hexapeptide-12 gels were localized in almost identical areas of their solution counterparts on the skin but with lower intensity. Coupled together, these observations provide a proof-of-concept on the use of OrbiSIMS to image cosmetic peptides in their sol and gel forms on the surface of human skin. In particular, this has been possible due to the high ion counts originating from the peptide Palmitoyl hexapeptide-12, which were relatively high if compared with other systems reported previously, such as palmitoyl-GHK.^33^ Finally, by doing an overlay of peptide ion signals (Figure 2Biii and Biv) with that from fatty acids and cholesterol sulfate (Figure 2B-i and B-ii), it was clear that peptide permeation and consequent penetration were not uniform on the SC layer, but it seemed localized, possibly intercalating within the SC components.^41^ Assembly and intercalation of Palmitoyl hexapeptide-12 within the skin was also demonstrated by Thioflavin T (ThT) staining, which is widely used as a fluorescent probe to monitor peptide aggregation and self-assembly into supramolecular structures. As shown in Figure 2C, large microscopic assemblies of Palmitoyl hexapeptide-12 were distributed on the uppermost layer of the SC, while smaller quantities of assembled peptides were observed within the SC, surrounding the skin corneocytes. Coupled together, these observations demonstrate that Palmitoyl hexapeptide-12, deposited on the surface of the skin as nonassembled peptide (or “sol” phase), starts to self-assemble into assembled fibers (or “gel” phase), visible both on the surface of the skin and intercalating within the SC components.

### 2.3 SRS Imaging of Stratum Corneum (SC) Treated with SAP

**Figure 3:**
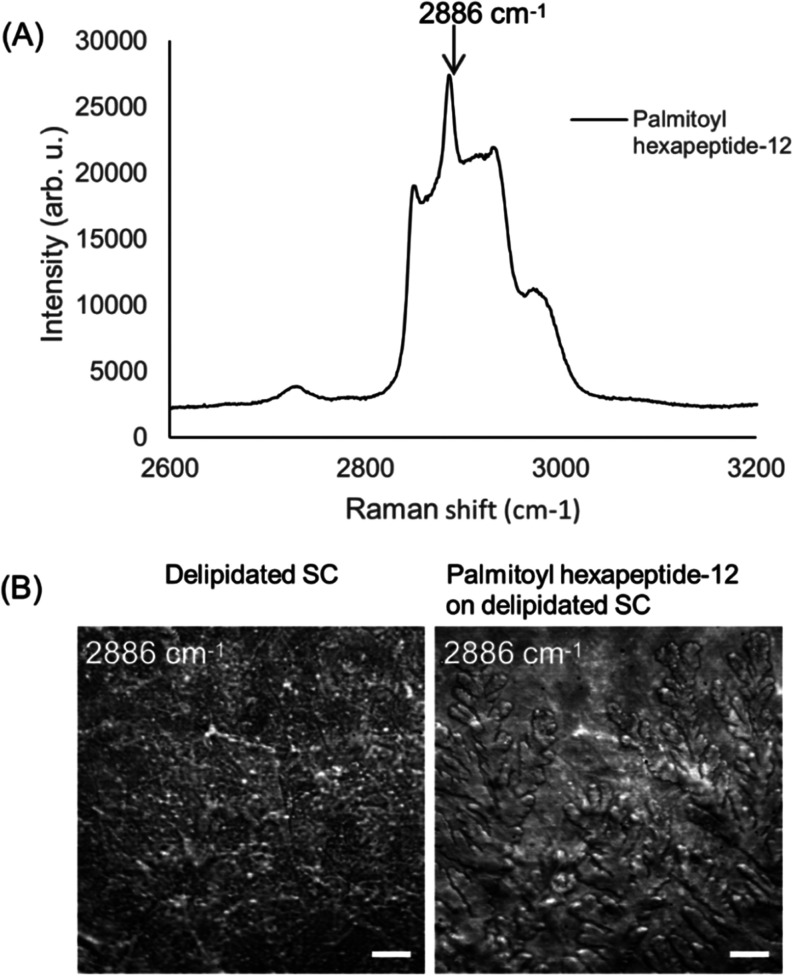
(A) The Raman spectrum of Palmitoyl hexapeptide-12 is in the high wavenumber region of the CH band. A prominent peak at 2886 cm^–1^ was selected for SRS imaging. (B) SRS images of a delipidated SC sample before and after treatment with the Palmitoyl hexapeptide-12 solution. Samples were dried before imaging. The morphology of the skin samples was visualized based on the SRS signals from the CH bonds. Scale bar: 20 μm.

SRS microscopy was used to characterize the biochemical composition of human skin SC samples, focusing on the distribution patterns and quantifications of lipids, proteins, and water under varied hydration states. The SRS technique was first applied to investigate the molecular distribution pattern of Palmitoyl hexapeptide-12 within the skin SC samples. As shown in Figure 3A, the Raman spectra of Palmitoyl hexapeptide-12 exhibit a pronounced peak at 2886 cm^–1^, which was utilized as a marker for imaging the compound on delipidated SC samples. Figure 3B showcases SRS images of the same delipidated SC sample before and after the treatment. It reveals a branched, aggregate pattern, indicating its gradual buildup and deposition over the skin. The comparative analysis of the topographical distribution pattern of the compound is pivotal, as it may also reflect the varying mechanisms through which it interacts with and penetrates the skin, highlighting the strength of SRS imaging in assessing the suitability and effectiveness of skincare treatments. This result demonstrates that SRS imaging can be used for visualizing and understanding the distribution and interaction of chemical compounds with skin tissues.^41^ Future studies could explore the deconvolution of amide I, II, and III peptide bands with high spectral resolution SRS imaging to investigate the secondary structure of proteins and gain deeper insights into sol and gel distributions.^42^

**Figure 4:**
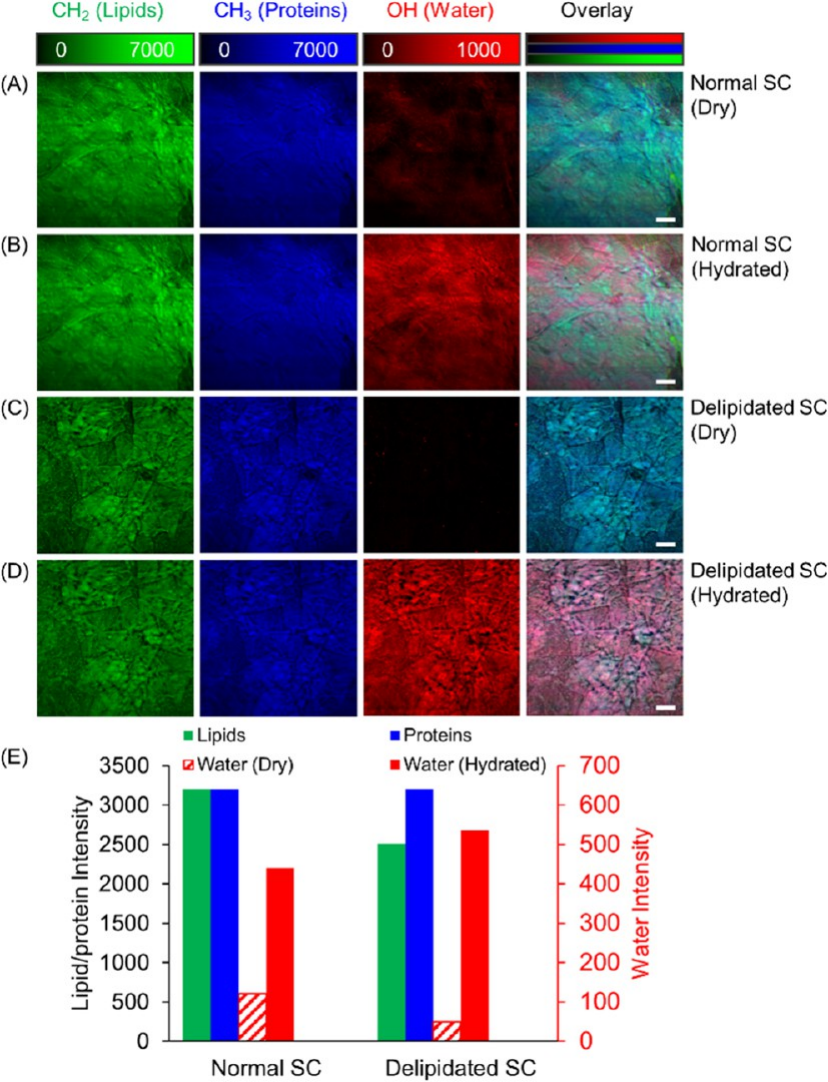
SRS imaging of SC samples. (A) Dry normal SC, (B) hydrated normal SC, (C) dry delipidated SC, and (D) hydrated delipidated SC. Green: SRS images at 2854 cm^–1^, attributed to the CH_2_ chemical bond vibration, represent total lipids. Blue: 2930 cm^–1^ for CH_3_ bonds, representing total proteins. Red: 3300 cm^–1^ for OH bonds, representing water distribution. (E) Averaged intensities of lipids, proteins, and water in the samples. Scale bar, 20 μm.

We used SRS microscopy to image and quantify the molecular components of stratum corneum (SC) samples under different hydration states.^43^ Total lipids (pseudo color green), proteins (blue), and water (red) content were imaged with SRS microscopy at 2854, 2930, and 3300 cm^–1^, which are the vibrational frequencies of CH_2_, CH_3_, and OH chemical bonds, respectively.^44,45^ We find that a normal SC sample in the dry state contains very little bound water (Figure 4A). After the same sample was hydrated with a humidifying air flow in a small enclosed chamber for 20 min, a notable increase in the water content was observed (Figure 4B). A similar increase of the water content from dry to hydrated states was found in a delipidated sample (Figure 4C and D). Figure 4E quantifies these observations, providing a direct comparison of lipid, protein, and water average intensities across the different SC samples and conditions. The data exhibit a discernible decrease in lipid content for the delipidated SC samples, with a concomitant decrease in the hydrating capacity, as evidenced by the lower red intensity levels under the dry condition. This quantitative evidence confirms the vital role of lipids in maintaining the SC’s capacity to retain water and highlights the potential detrimental effects of lipid loss on skin hydration and barrier function.^46−48^

**Figure 5:**
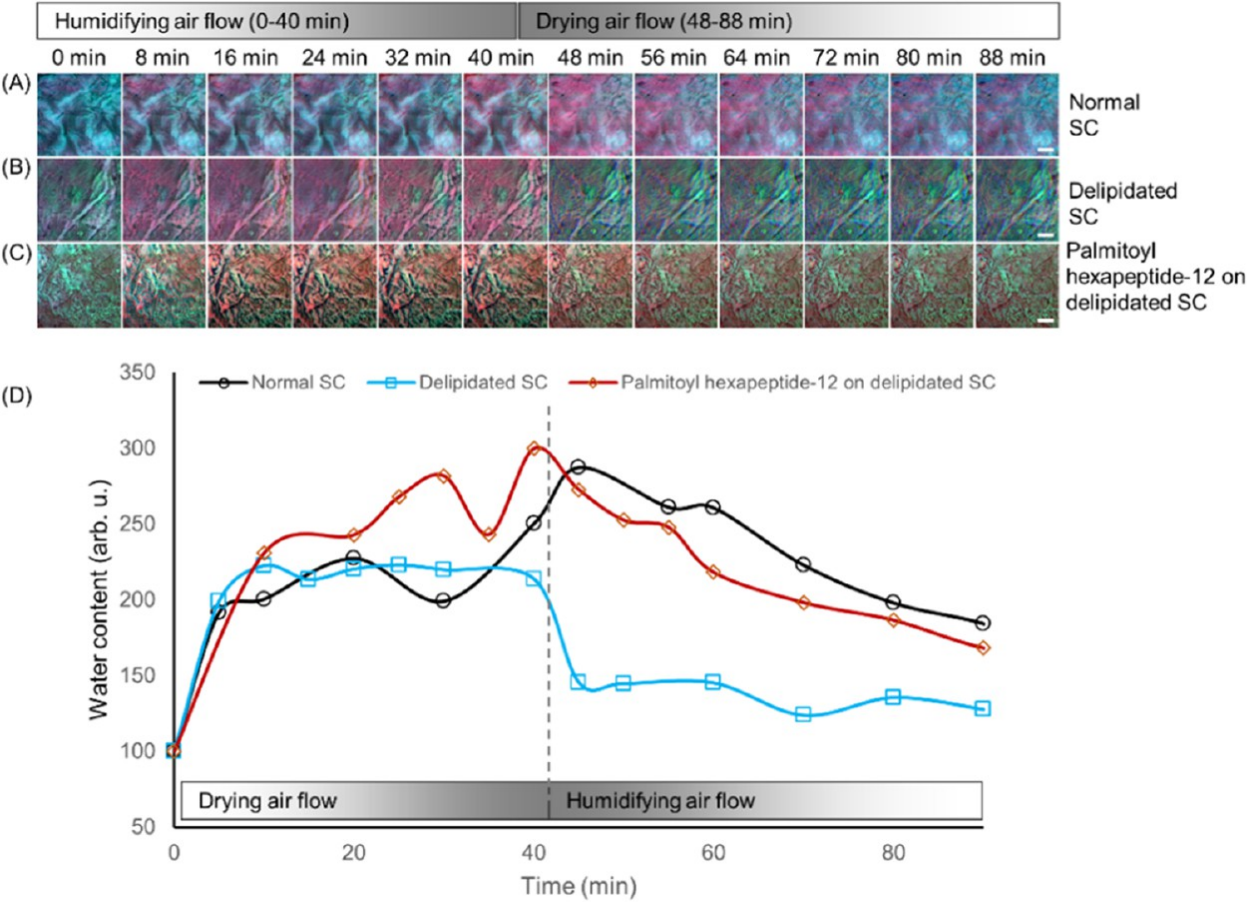
Time-lapse SRS imaging of the water content in SC samples enclosed in a chamber. The chamber was first purged with humidifying air flow for 40 min (flow rate: ∼1.0 L/min) and then purged with drying air flow for 50 min (flow rate: ∼0.5 L/min). (A) Normal SC, (B) delipidated SC, and (C) delipidated SC treated with the Palmitoyl hexapeptide-12 (0.45% w/v). Delipidated SC showed a much faster water loss under the drying flow through the chamber. (D) Application of Palmitoyl hexapeptide-12 treatment reduced water loss significantly on the delipidated SC samples. Green: lipids, blue: proteins, and red: water. Scale bar, 20 μm.

Figure 5 elucidates the dynamics of water content in stratum corneum (SC) samples using time-lapse SRS imaging in a controlled chamber environment.^49^ Through this approach, we investigated the hydration and subsequent dehydration processes in SC samples, capturing a comprehensive view of water content fluctuation over time. Figure 5A displays a normal SC sample undergoing a 40 min humidifying air flow, followed by a 50 min drying air flow, illustrating a gradual increase in water content during humidification and a steady decrease upon drying. This normal SC’s water content serves as a baseline in this study.^50^ In contrast, a delipidated SC sample (Figure 5B) showed an accelerated loss of water under drying conditions, substantiating the hypothesis that lipids play a crucial role in water retention within the skin SC.^51^ The rapid water loss observed in the delipidated SC underscores the importance of the lipid matrix for barrier function and hydration maintenance. Exploring potential therapeutic interventions, Figure 5C shows delipidated SC samples treated with a Palmitoyl hexapeptide-12 solution. Notably, the treatment demonstrated a retardation of water loss compared to the untreated delipidated SC. The Palmitoyl hexapeptide-12 treated SC nearly mimics the hydration retention profile of normal SC, suggesting a restoration of barrier function. The quantitative plot in Figure 5D captures these observations, presenting water content changes over time across all three sample conditions. The graphical representation highlights the striking contrast between the rapid dehydration of delipidated SC and the mitigating effects of Palmitoyl hexapeptide-12 treatment. This data not only confirms the vital role of lipids in maintaining SC hydration but also demonstrates the potential of the compound to recuperate the skin’s natural barrier and hydration retention capabilities.^52^

### 2.4 Biomechanical Assessment of Skin Barrier Function Following Peptide Topical Treatment

**Figure 6:**
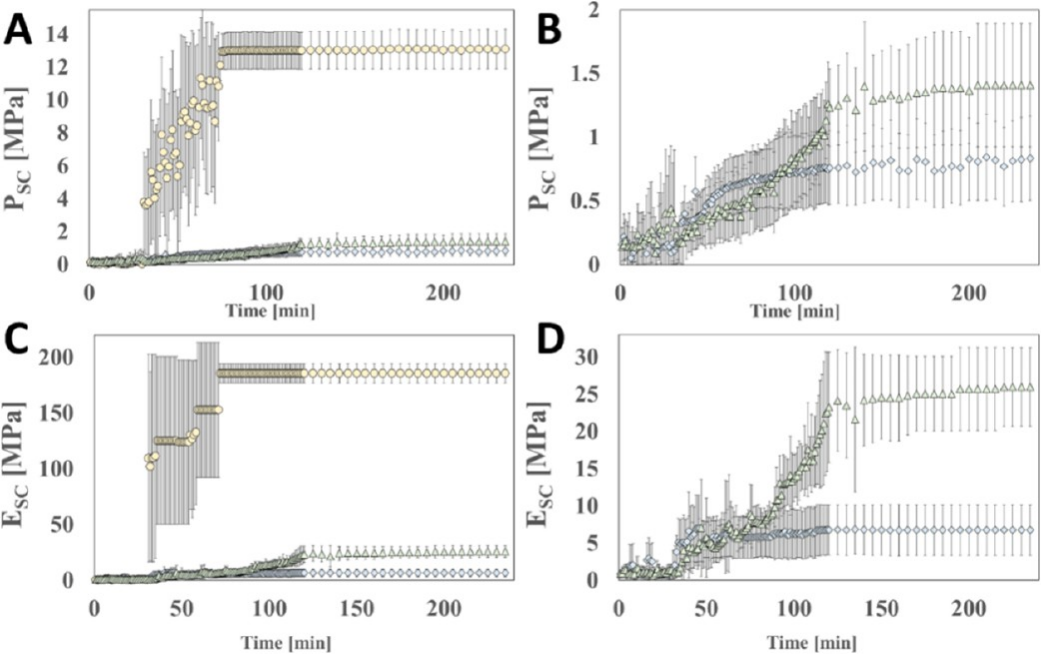
SC drying mechanics. Scatter plots of averaged SC contractile drying stress P_SC (A, B) and elastic modulus E_SC (C, D) measurements plotted against drying time for samples treated with DIW (yellow solid circle), 5% GLY (blue solid diamond), and Palmitoyl hexapeptide-12 (green solid triangle). Planes B and D do not include DIW treatment. Error bars denote a standard deviation of 3 ≤ *n* ≤ 5.

Characterization of the barrier function of human stratum corneum (SC) with different treatments was performed using a high-throughput mechanical characterization method.^53,54^ The method assesses the health of the stratum corneum (SC) barrier function by quantifying its moisture retention capacity, as determined by observing the SC’s response to drying deformations under low relative humidity (% R.H.) conditions. In comparison with human SC tissue containing healthy lipid compositions, lipid-depleted SC tissue exhibits notably more rapid increases in the drying rate, the onset of drying stresses (P_SC_) that can drive tissue fracture, and tissue stiffness (E_SC_) during drying. This highlights the reduced water-holding capacity of the tissue in low humidity environments.^53,54^ Commonly used humectants such as glycerol at low concentrations (2–5%) improve water retention in lipid-depleted SC, reducing the tissue’s elastic modulus and drying stress magnitudes.^53,54^ While a variety of moisturizers are available on the market, including humectants and occlusives, in this study, the impact of Palmitoyl hexapeptide-12 in restoring the barrier function of lipid-depleted SC tissue is completed and compared with both deionized water (DIW) and 5% glycerol (GLY) controls. Although comparisons could be made with numerous moisturizers, the Palmitoyl hexapeptide-12 treatments studied here are readily absorbed into the skin in a fashion similar to that of humectants. In comparison with human SC tissue containing healthy lipid compositions, lipid-depleted SC tissue exhibits notably more rapid increases in drying rate, the onset of drying stresses that can drive tissue fracture, and tissue stiffness during drying. This highlights the reduced water-holding capacity of the tissue in low-humidity environments.^53,54^ Results reveal Palmitoyl hexapeptide-12 enhances the barrier function of lipid-depleted SC samples, as the treatment significantly reduces stiffness and drying stress observed in the low relative humidity conditions. Figure 6 shows the dynamic changes in elastic modulus and drying stress of fully hydrated and lipid-depleted SC samples treated with different formulations drying in a low humidity (25 ± 2% R.H.) environment after initially being equilibrated to a high humidity (100% R.H.) for 24 h. Humidity control is achieved using a microscope-mounted environmental control system (MEC). Figure 6A,C indicates that DIW-treated delipidated SC samples (white solid circles) experience a higher magnitude of contractile drying stresses (*P*_SC_) and elastic moduli (*E*_SC_) during drying compared to the other glycerol or water treatments.

**Figure 7:**
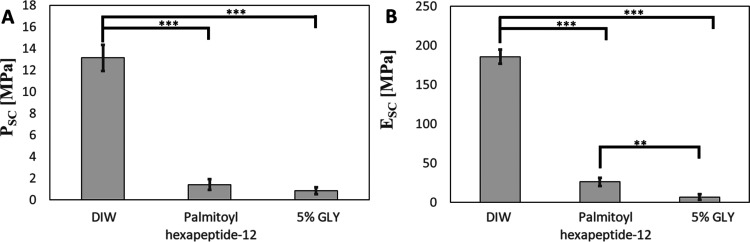
Final SC contractile drying stress, P_SC, and elastic modulus, E_SC. Bar graph of averaged final (*t* = 235 min) SC contractile drying stress P_SC (A) and elastic modulus E_SC (B) for samples treated with DIW, 5% GLY, and Palmitoyl hexapeptide-12. Error bars denote a standard deviation of 3 ≤ *n* ≤ 5. *, **, and ***, respectively note significant levels of *p* < 0.05, *p* < 0.01, and *p* < 0.001.

Between the switch to the low humidity condition (*t* = 30 min) and end of the drying (*t* = 235 min), DIW-treated SC samples experience at least a 10-fold increase in their drying stresses and elastic moduli when compared to the other treatments in the study, as shown in Figure 7A,B. The results reveal that DIW-treated SC samples experience near instantaneous water loss when transitioned to the low humidity environment, reaching an average final elastic modulus of *E*_SC_ = 185.7 ± 8.8 MPa and an average final drying stress of *P*_SC_ = 13.1 ± 1.2 MPa. These results indicate poor barrier restoration as observed in prior studies.^53,54^ As can be seen in Figure 7A, the average final drying stress of 5% GLY treated samples (*P*_SC_ = 0.8 ± 0.3 MPa), Palmitoyl hexapeptide-12 treated samples (*P*_SC_ = 1.4 ± 0.5 MPa) have statistically significant differences from DIW-treated samples (all *p* < 0.001). There are no statistically significant differences between final drying stresses observed between 5% GLY and Palmitoyl hexapeptide-12. Figure 7B indicates that the average final elastic modulus of 5% GLY treated samples (*E*_SC_ = 6.8 ± 3.4 MPa), Palmitoyl hexapeptide-12 treated samples (*E*_SC_ = 26.0 ± 5.3 MPa), have statistically significant differences from DIW-treated samples (all *p* < 0.001). Additionally, significant differences can be observed between the final elastic modulus of Palmitoyl hexapeptide-12 treated samples, as well as significant differences between the final elastic modulus of Palmitoyl hexapeptide-12 treated samples and 5% GLY treated samples (*p* < 0.01). The drying mechanics of lipid-depleted SC treated with Palmitoyl hexapeptide-12 compared to DIW highlights improved barrier function attaining parity with optimal moisturizers containing 5% glycerol.

### 2.5 *In Vitro* Characterization of Peptide-ECM Co-assembly

#### 2.5.1 Gelling Properties upon Co-assembling

**Figure 8:**
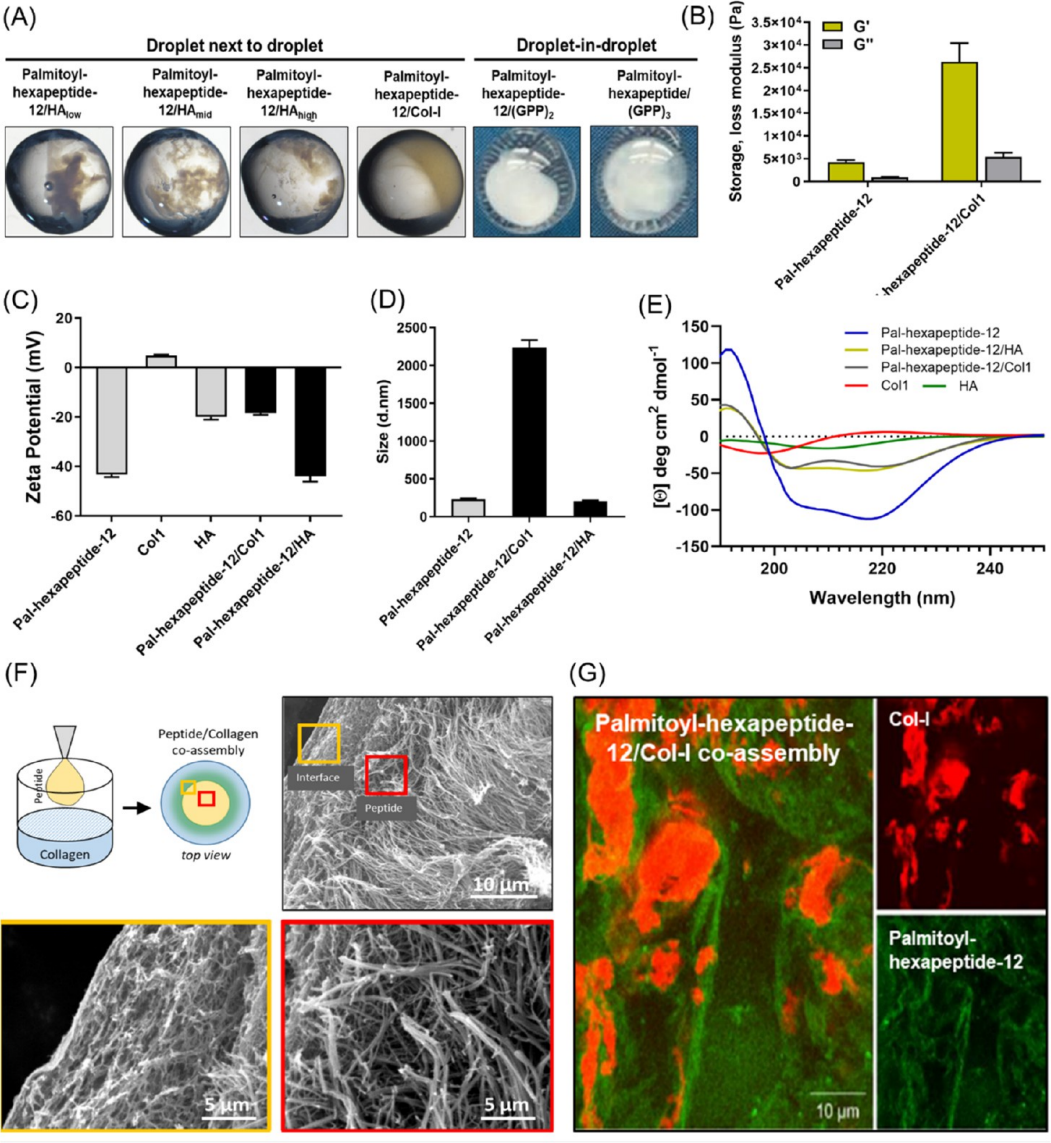
Co-assembly of palmitoyl hexapeptide-12 with human skin ECM components. (A) Gelling properties upon co-assembling of Palmitoyl hexapeptide-12 with hyaluronic acid (HA_low_: 8–15 kDa, HA_mid_: 130–150 kDa and HA_high_: 750–1000 kDa), collagen type I and collagen-derived peptide ((GPP)2: GPPGPP and (GPP)3: GPPGPPGPP). (B) Rheological properties of co-assembled Palmitoyl hexapeptide-12-collagen type I gels. (C) Zeta potential, (D) Dynamic light scattering (DLS), and (E) circular dichroism analysis of Palmitoyl hexapeptide-12 co-assembling with collagen type I and HA molecules. (F) Scanning electron microscopy shows a characteristic diffusion-driven interface forming between co-assembling Palmitoyl hexapeptide-12 and collagen type I droplets. (G) Confocal microscopy of co-assembling Palmitoyl hexapeptide-12 and collagen type I solutions.

A series of *in vitro* tests were conducted to assess the interactions of PAs with the ECM components. As an initial assessment of PA/ECM co-assembly, we tested Palmitoyl hexapeptide-12 to co-assemble with ECM components present in the human skin. We have previously demonstrated the capability of PAs to co-assemble with proteins and ECM components, such as elastin-like polypeptides and HA, to create multi-component membranes and sacs for tissue engineering applications.^55^ In the case of skin, the native ECM possesses a large number of structural and signaling components, including collagen, elastin fibers, proteoglycans, and glycosaminoglycans (GAGs). In particular, collagen type I is the most abundant protein accounting for skin structural integrity, while HA is responsible to retain large amounts of water and provide skin moisture and elasticity.^56^ Due to their importance in the mechanics of native skin, we, therefore, focused our preliminary test on the co-assembly of topical peptides with collagen I (Col-I) and HA. To do so, stereoscopic microscopy was initially employed to observe the liquid–liquid interface formed when droplets of peptides were placed in direct contact with droplets of ECM component solutions (Figure 8A). Upon contact of Palmitoyl hexapeptide-12 with collagen I and HA, an interface spontaneously formed, triggering the formation of co-assembled structures. Interestingly, the interaction of Palmitoyl hexapeptide-12 with collagen I and HA led to dark co-assembled structures, suggesting immediate interaction. Moreover, the co-assembly of Palmitoyl hexapeptide-12 with HA molecules occurred at multiple HA molecular weights, suggesting strong interactions between the two components. To further investigate the interaction of Palmitoyl hexapeptide-12 with collagen I, we also injected Palmitoyl hexapeptide-12 into water solutions containing the consensus collagen-derived triple-helix motif GPP.^57^ Also in this case, a full integration of peptides and collagen motifs into robust hydrogel networks was observed (Figure 8A). Based on this preliminary visual assessment, we hypothesized the formation of dense and strong gelatinous networks for Palmitoyl hexapeptide-12-ECM assemblies. To prove that, we also performed oscillatory rheology on peptide gels and peptide-ECM co-assembled gels. As shown in Figure 8B, coassembled Palmitoyl hexapeptide-12/Col-I resulted in significantly stiffer hydrogels (nearly 5-fold increase) than Palmitoyl hexapeptide-12 only. Taken together, these results demonstrated that Palmitoyl hexapeptide-12 can co-assemble with ECM components of the skin, such as collagen I and HA resulting in strong co-assembled structures.

#### 2.5.2 Supramolecular Characterization of Co-assembled Structures

To investigate the supramolecular coassembly of Palmitoyl hexapeptide-12 with HA and collagen molecules, we used a combination of zeta potential, dynamic light scattering, and circular dichroism measurements. Indeed, molecular co-assembly is normally triggered by complexation of molecules with opposite charges, leading to supramolecular structures with increasing molecular size after coassembly.^58^ As shown in Figure 8C, before co-assembly, Palmitoyl hexapeptide-12 and HA exhibited a negative charge, while the collagen was positively charged at pH 7. After co-assembly, the Palmitoyl hexapeptide-12/Col-I and Palmitoyl hexapeptide-12/HA co-assembled structures both showed a negative charge suggesting a strong electrostatic interaction with Palmitoyl hexapeptide-12. During co-assembly, opposite charges often trigger a strong co-assembling reaction that generates larger aggregates.^58^ To study this aspect, we employed dynamic light scattering to assess the size of the peptide before and after co-assembly. Palmitoyl hexapeptide-12 showed a significant increase in size upon co-assembly with collagen I, resulting in larger aggregates, while no evident differences were observed with HA molecules (Figure 8D). Furthermore, in terms of secondary structure, Palmitoyl hexapeptide-12 showed an increase in the α-helix component after coassembling with collagen, suggesting the creation of more ordered co-assembled structures upon contact of the two systems (Figure 8E). Multiple peptide-based systems have shown a relationship between the final mechanical properties of peptide hydrogels and their secondary structures.^59,60^ In this case, a more ordered secondary structure observed for Palmitoyl hexapeptide-12/collagen type I assemblies resulted in self-supporting and elastic peptide-ECM hydrogels. Taken together, these observations highlight how the coassembling mechanism of Palmitoyl hexapeptide-12 with ECM components is a supramolecular process starting at the nanoscale and having an effect on macroscale features.

#### 2.5.3 Morphological Features of Co-assembled Structures

After having assessed the gelling properties and the supramolecular assembly, we further characterized the morphology of Palmitoyl hexapeptide-12-ECM co-assemblies at the microscale using scanning electron microscopy (SEM) and fluorescence imaging. The SEM observations revealed a gradient structure going from the interface of collagen and peptides toward the inner structure of the coassemblies (Figure 8F). These gradient structures are characteristic of diffusion phenomena occurring at the interface of peptide solutions coassembling with proteins, and our group has reported similar structures for PAs interfacing with HA,^55^ ELP,^23^ keratin, and collagen.^27^ Under fluorescence microscopy, it was evident the co-assembly of Palmitoyl hexapeptide-12 and collagen domains, with collagen type I forming individual patches (red) within a fibrillar peptide mesh (green) (Figure 8G). Morphological studies revealed a full integration of the topical peptide Palmitoyl hexapeptide-12 with collagen I. These results highlight how peptides could be used as nanotools to target ECM molecules present in the skin and co-assemble with them for skincare and cosmetic purposes.

### 2.6 Histological Analysis of Interaction of Palmitoyl Hexapeptide-12 with Elastin

**Figure 9:**
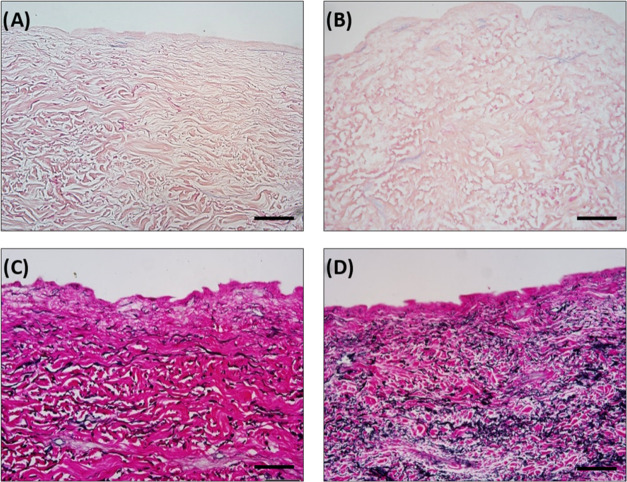
Representative H&E images and Van Gieson images of human skin before and after peptide treatment. (A) *Ex vivo* decellularized human skin treated with PBS as the control. (B) *Ex vivo* decellularized human skin treated with the Palmitoyl hexapeptide-12. Both images display a lack of blue/purple nuclear stain indicating effective decellularization. (C) *Ex vivo* decellularized human skin treated with PBS as the control. (D) *Ex vivo* decellularized human skin submerged in peptide solution showing a robust increase in elastin staining after 5 days of treatments. All images captured at 20× magnification. Scale bar: 50 μm.

Working alongside collagen, elastin plays a key role in skin tone and elasticity.^56^ To check the interactions of Palmitoyl hexapeptide-12 with elastin, histological sections and staining of human skin were performed. In particular, decellularization of *ex vivo* human skin was performed to preclude any potential biogenesis of new elastin fibers, while maintaining the existing extracellular matrix. H&E staining of peptide-treated decellularized dermal tissue confirmed that each treatment was successfully decellularized (Figure 9A,B). The faint blue staining observed in some of the images was not indicative of nuclei, as there was no defined nucleus-like morphology. At this stage, the ability of the skin to synthesize elastin or biogenesis can be presumed to be absent, leaving peptide co-assembly as the only relevant source of additional fibers. To test this hypothesis, elastin Van Gieson staining was performed. As shown in Figure 9C,D, the elastin Van Gieson staining demonstrated that the Palmitoyl hexapeptide-12 is binding to existing fibers denoted by the noticeable increase in elastin fiber stain (black signal) that was observed in the skins submerged in peptide solution (Figure 9D) compared to the skins submerged in phosphate-buffered saline (PBS) (Figure 9C). Since the skin lost its ability to synthesize elastin fibers, this evidence suggests the mechanism of action of these peptides may be, in part, due to coassembly properties of the cosmetic peptide with the ECM components of the human skin.

## 3 Conclusion

The ability of Palmitoyl hexapeptide-12 peptides to penetrate and assemble within the human skin across both superficial and deeper layers was demonstrated using a combination of OrbiSIMS, *in vitro* gelling tests, and spectroscopy analyses. This study marks the first application of OrbiSIMS to identify and track the different stages of supramolecular assembly of an SAP within human tissue with exceptional mass accuracy. These findings not only advance our understanding of peptide penetration mechanisms but also pave the way for the design of innovative skincare solutions tailored to enhance skin penetration. Alongside penetration and assembly, the application of SRS microscopy enabled the analysis of biochemical composition and hydration dynamics of the human skin, elucidating the critical role of lipids in maintaining barrier function and water retention. Palmitoyl hexapeptide-12 treatment, measured via mechanical analysis, enhances the barrier function of lipid-depleted SC, with a significant reduction in skin stiffness and drying stress. This result further underscores the potential of Palmitoyl hexapeptide-12 to restore skin hydration capacity, offering new opportunities for skincare development. Moreover, through histology, we confirmed the co-assembly and integration of Palmitoyl hexapeptide-12 with elements of the human skin, in particular with elastin fibers involved in skin’s elasticity, as an *ex vivo* decellularized skin showed an increase in elastin fiber diameter compared to counterparts treated with PBS solutions. The combination of OrbiSIMS, gelling tests, SRS, histology, and mechanical testing provides an objective, label-free, and comprehensive battery of techniques to evaluate the efficacy and mode of action of Palmitoyl hexapeptide-12 in the human skin, across multiple skin layers. We envisage that our proof-of-concept approach could be applied to other tissue-penetrating cosmetic SAPs and that it will set the standards to progress the field of cosmetic science.

## 4 Materials and Methods

### 4.1 Materials

Palmitoyl hexapeptide-12 (purity >98%) was purchased from Biomatik (ON, Canada). Collagen type I from rat tail (product code: C3867 1-VL), and HA with low, middle, and high molecular weights *M*_w,low_ = 8–15 kDa, *M*_w,mid_ = 130–150 kDa and *M*_w,high_ = 750–1000 kDa (product codes: 40583, 75043, and 53163) were purchased from Sigma-Aldrich.

### 4.2 Peptide Solutions and Gel Preparation

Topical peptide solutions at 1 wt % were prepared by dissolving 10 mg of lyophilized peptide powder into 800 μL of deionized water and by adjusting pH to 7 using dropwise additions (5 μL steps) of 1 M NaOH. The remaining deionized water was added to reach a final volume of 1 mL. For the gel preparation, peptide hydrogels with final volumes of 1 mL were obtained from peptide solutions by adding NaCl powder and remaining deionized water to a final concentration of 3 wt %. Successful gelation was confirmed using the tilting tube test, i.e., samples were classified as a “sol” when the sample flowed freely, while it was classified as “gel” when the sample was self-supporting upon vial inversion.

### 4.3 *In Vitro* Co-assembly

To enable real-time observations of the co-assembly mechanism occurring between the topical peptides and the ECM molecules, we used an interface and a droplet-in-droplet experimental setup. In the interface setup, two 5 μL-droplets of topical peptide solution and ECM molecule water solutions were placed side-by-side on a polydimethylsiloxane (PDMS) substrate. For the droplet-in-droplet setup, 10 μL of 2% peptide solutions were injected into a 20 μL droplet of 2% ECM molecules in water solutions. Droplets in contact started to form new structures at both liquid–liquid and liquid-in-liquid interfaces. Droplets were imaged 10 min after contact with an optical microscope.

### 4.4 Circular Dichroism (CD)

CD was used to analyze the secondary structure of topical peptides before and after co-assembling with ECM molecules. CD measurements were carried out on a Chirascan CD Spectrometer (Applied Photophysic Limited, U.K.) at room temperature. All samples were prepared at 0.1 wt % in deionized water. For co-assemblies, peptides and ECM molecules were mixed in 1:1 v/v ratio and left to react for 1 h before testing. A quartz cuvette with 0.1 cm path length was used for the measurements and CD spectra were obtained by signal integrating 10 scans, from 190 to 260 nm at a speed of 50 nm min^–1^. Data were processed by a simple moving average and smoothing method.

### 4.5 Zeta Potential (ζ)

To illustrate the co-assembling mechanism of the topical peptides with the ECM molecules, their zeta potentials (ζ) were measured on Zetasizer (NanoZS ZEN 3600, Malvern Instruments, Worcestershire, U.K.) at room temperature. Peptides were prepared in deionized water at a concentration of 0.05 wt %, while ECM molecules were used at 2 wt %. The samples were equilibrated for 30 min at room temperature prior to the measurement of zeta potential and loaded with a syringe into a folded capillary Malvern disposable cuvette (DTS1070, Malvern Panalytical, U.K.). Molecules were used at their original pH, while the pH of peptides was adjusted to pH 3, 7, and 10 using 1 M HCl or 1 M NaOH to evaluate the surface charge of peptides at acidic, neutral, and basic pH.

### 4.6 Dynamic Light Scattering (DLS)

DLS was performed to measure the particle sizes of topical peptides before and after co-assembling with ECM molecules, as it is expected that the co-assembling reaction will generate larger aggregates. The topical peptides were dissolved in deionized water at concentrations of 0.05 wt %, while molecules were used at 2 wt %. The two solutions were mixed in a 1:1 volumetric ratio and loaded with a syringe into a folded capillary Malvern disposable cuvette (DTS1070, Malvern Panalytical). The particle sizes of each solution were measured using a Zetasizer machine (NanoZS ZEN 3600, Malvern Instruments, U.K.). Samples were equilibrated for 10 min at the desired temperature before measurements.

### 4.7 Oscillatory Rheology

Rheological measurements were performed using an MCR-302 Modular Compact Rheometer (Anton Parr). Topical peptide gels were prepared at 1 wt %, while in co-assembling systems final gels were obtained by mixing 1 wt % peptides with 0.4 wt % collagen. Hydrogels were mounted on the rheometer plate and tested with an 8 mm parallel plate geometry and a measurement gap size of 0.5 mm. Amplitude sweep measurements were performed at a constant frequency (1 Hz) with strain ranging from 0.01 to 100% strain.

### 4.8 Scanning Electron Microscopy (SEM)

SEM was used to investigate the microstructure properties of co-assembled Palmitoyl hexapeptide-12 with collagen. For the co-assembling structures, 1% topical peptides were mixed with 0.4 wt % rat tail collagen in 1:1 v/v ratio. The resulting topical peptide-collagen co-assembled gels were fixed at room temperature with 4% paraformaldehyde solution (PFA) for 20 min, washed of excess PFA with deionized water, and dehydrated using a series of increasing ethanol concentrations (20, 50, 70, 90, 96, and 100%) for 5 min each. All samples were then subjected to a critical point dryer (Leica EM CPD300, Leica, Wetzlar, Germany), mounted on SEM stubs covered by conductive carbon adhesive tape, and sputter-coated with gold (10 nm thick coating) before imaging with an Inspect Q600 (FEI Comp, Eindhoven, Netherlands).

### 4.9 Confocal Fluorescence Microscopy

The interaction and co-assembly of topical peptides with collagen type I were evaluated using confocal fluorescence microscopy (TCS SP2, Leica Microsystems, Germany). Before imaging, 1 mL of 1 wt % Palmitoyl hexapeptide-12 water solution was mixed with 2 μL of 0.1 wt % ThT (green dye), while 1 mL of 0.4 wt % collagen water solution was mixed with 2 μL of 0.1 wt % Alexa Fluor-647 NHS Ester (red dye). All solutions were incubated for 20 min at 30 °C and protected from direct light. Images were acquired at laser wavelengths of 440 and 650 nm which correspond to the excitation wavelength of ThT and Alexa Fluor-647 dyes, respectively.

### 4.10 Human Skin Acquisition and *In Vitro* Permeation

A full-thickness *ex vivo* human skin sample was purchased from BioIVT (product name: ASTERAND Human Skin Normal). Skin tissue was obtained from the abdomen of a 58 years old, nonsmoker Caucasian female donor was removed during cosmetic surgery. The tissue was frozen immediately postsurgery, shipped, and stored at −20 °C prior to use. Once defrosted, circular *ex vivo* human skin was cut and mounted, dermal side down, between a donor and receptor chamber in a Franz-type static diffusion cell setup with an exposed surface area of 1.1 cm^2^ and a receptor compartment volume of 3 mL. The receptor compartment was filled with phosphate-buffered saline (pH 7.5) and an infinite dose of Palmitoyl hexapeptide-12 solution (100 μL of 1% peptide per cm^2^) was applied via the donor compartment. Sink conditions were maintained throughout the experiments. Skin samples were exposed to formulation for 8 h in a water bath set 36.5 °C, which maintains a temperature of 32 °C. After 8 h, the Franz cell was dismantled, and the excess solution was removed with a dry sponge. The skin was wiped with a sponge soaked in Teepol solution (3% v/v) and then dehydrated under vacuum at room temperature before loading into the Hybrid-SIMS instrument for analysis.

### 4.11 OrbiSIMS Single-Beam Depth Profile Analysis of *Ex Vivo* Skin

Human skin samples were loaded into the ToF-SIMS machine coupled with an Orbitrap mass spectrometer and the apparatus was set up as described previously.^33^ In brief, skin samples were first introduced into the airlock on the cryostage of the instrument. This was flooded with argon gas, and the cooling process was initiated. Once the stage reached −80 °C, the vacuum pump was initiated, which accelerated the cooling process and brought the stage, and consequently skin, temperature down to −170 °C. During both the vacuum transfer and the analysis, the sample was maintained at this temperature using a closed-loop liquid nitrogen pumping system (IONTOF GmbH), which allowed stable cryooperation for more than 24 h with a single dewar filling. For the depth profile analysis, an Ar3000+ analysis beam with an energy of 20 keV and a diameter of 20 μm was used as the primary ion beam. The duty cycle of the beam was set to 4.4%, and the gas cluster ion beam (GCIB) current was 250 pA. The depth profile was run on an area of 200 × 200 μm using a sawtooth raster mode with crater size 280 × 280 μm. The cycle time was set to 200 μs. Optimal target potential was averaged at approximately ±384.5 V. Depth profiles were collected in both positive and negative polarity, in the mass range of 75 to 1125 *m*/*z* and the injection time was set to 500 ms. Mass-resolving power was set to 240,000 at 200 *m*/*z*. All data analysis was carried out using Surface Lab 7.1 (IONTOF GmbH). Depth profiles were compressed using a running average method (100 data points), where the individual data points represent the secondary ion intensities for a selected mass peak at subsequent depths of the sample. Orbitrap data were acquired by using a ThermoFisher Orbitrap HF mass spectrometer. Assignments were determined by accurate mass within a 3 ppm error of the calculated mass.

### 4.12 High-Resolution Ion Imaging

High-resolution images of the skin surface were acquired using a 20 keV Ar1700+ cluster ion beam with a primary ion current of 9 pA. The beam size was around 2 μm in diameter, but the final pixel size was set to 4 μm to reduce image acquisition time. The primary ion dose was distributed equally across each pixel area by restoring the beam in a 10 × 10-pixel microraster. Four imaging scans were acquired from a field of view area of 300 × 300 μm^2^ leading to a total primary ion dose density of 2.65 × 10^10^ ions/cm^2^. Charge compensation was performed by flooding the sample with 20 eV electrons. The mass resolution was set to 240,000 (at *m*/*z* 200) and a fixed injection time of 500 ms was used.

### 4.13 SIMS-MPF Software

Molecular formula prediction over the negative ion depth profile OrbiSIMS data set acquired was performed using SIMS-MPF software, as described previously.^33^ After performing a peak search using the SurfaceLab software (IONTOF, GmbH) (minimum count = 70,000), assignments of characteristic skin components, such as C*_n_*H*_n_*O^2–^ (fatty acids), C*_n_*H*_n_*O^6–^ (triglycerides), C*_n_*H*_n_*O*_n_*N^1–^ (ceramides), C*_n_*H*_n_*O*_n_*S^1–^ (sulfates), and C_<6_H_<20_N_<4_O_<3_^–^ (amino acids), where *n* = any integer value, were made from the output data tables. All assignments were made with <2 ppm mass deviation. Molecular assignments were then verified by comparing against the LIPID MAPS Structure Database (42) and/or previous literature.

### 4.14 Thioflavin T (ThT) Staining

ThT staining was used to image the assembly and intercalation of Palmitoyl hexapeptide-12 fibers within the skin surface. Before staining, individual layers of the SC were isolated from human skin using a tape-stripping method developed by Starr and colleagues.^33^ In particular, using an adhesive double-sized tape, three consecutive tape strips were taken to remove individual corneocyte layers from the skin, with the first strip representing the uppermost layer of the SC. Following tape-stripping, the second side of the tape was adhered on a glass slide and the skin layers were exposed to a solution of 1 wt % Palmitoyl hexapeptide-12 for 10 min. During incubation, 50 μM of ThT aqueous solution were added to the peptide solution (peptide: ThT ratio of 9:1) and left to interact for 30 min at room temperature. After incubation, excess solution was removed with Whatman paper, and glass slides were washed with deionized water.

### 4.15 Stratum Corneum Sample Preparation

Full-thickness skin received from a cadaveric source (96 M, left abdomen) was used for all drying studies. In detail, full-thickness 96 yrs. male abdominal skin tissue was received from ConnectLife (Buffalo, NY) within 24 h of elective surgery. An exempt approval (3002–13) was obtained to perform research using deidentified tissue samples pursuant to the Department of Health and Human Services (DHHS) regulations, 45 CFR 46.101:b:4. SC was isolated using a standard heat bath and trypsin technique.^53,61,62^ The skin was sourced from this photoprotected anatomical region to limit the influence of ultraviolet light-based photoaging.^63−64,65,66^ The stratum corneum (SC) layer of the skin was isolated and delipidated. Once isolated, SC sheets were placed on plastic mesh, rinsed in deionized water, and dried for 48 h at room temperature and humidity. Details of the SC isolation and lipid depletion procedures are described in prior studies.^56,57,67^ Consistent diameter sample geometries were achieved by cutting samples with a hole punch (*R* = 3.1 ± 0.25 mm), Harris Uni-Core, Redding, CA. A J-shaped mark was made on the topside of each sample using an indelible marker to distinguish the superior and inferior sides of the SC. SC samples were agitated for 30 min in a fluorescent marker bead (505/515 nm, 1 μm diameter, carboxylate-modified, Molecular Probes, Invitrogen, Grand Island, NY) solution diluted in deionized water (90 μL in 15 mL DI water). The SC samples were then laminated onto elastomer-coated coverslips. The methodology for the preparation and curing of the elastomer-coated coverslips, and the lamination of the samples onto the substrate are also described in prior studies.^56,57^ The stiffness^68^ of the elastomer film on the coverslips is lower than the reported stiffness of the epidermal tissue, mimicking the softer underlying tissue beneath the SC.^69^ It is this difference in stiffness that allows for the energy relationship between the deformation of the SC and the elastomer to be leveraged to calculate the elastic modulus and drying stress values. Following lamination, SC samples were dried in ambient conditions (20 °C, 40% R.H.) for 60 min to allow evaporation of residual water between the SC samples and the elastomer-coated coverslip,^56,57^ resulting in adhesion of the sample to the substrate without entrapped bubbles. Each coverslip was laminated with 3 ≤ *n* ≤ 5 SC samples.

### 4.16 Stratum Corneum Sample Treatment and Storage

Adhered SC samples and substrates were immersed upside down in a Petri dish containing 15 mL of different solutions. Solutions included deionized water (DIW), 5% glycerol (volume/volume) in deionized water (5% GLY), and 0.45% (w/w) Palmitoyl hexapeptide-12 in 1× phosphate-buffered saline (PBS, K813–500 ML, AMRESCO, Cleveland, OH). SC samples were exposed to treatments for 60 min to maximize treatment effects without damaging intercellular lipid lamellae.^56,57,70^ Samples were then removed from the treatment solution and stored in an environmental perfusion chamber with an internal humidity of 99% R.H. for 24 h to equilibrate.

### 4.17 Imaging In-Plane Drying Displacements

Once fully hydrated, SC samples were mounted in a microscope-mounted environmental control (MEC) system to minimize exposure to ambient humidity conditions. Transfer of SC samples to the MEC was done using a portable bespoke humidity chamber to minimize water loss prior to each experiment. Samples were placed in the MEC and exposed to the high humidity condition (100% R.H.) for the first 30 min of imaging, during which no in-plane sample contractility occured due to the SC samples remaining fully hydrated. The lack of contractile drying forces can be observed in Figure 1A,C. The MEC was transitioned to the low humidity condition (25 ± 2% R.H.) after 30 min for the remaining 205 min of imaging to induce contractile drying forces. Images were acquired every 60 s for the first 120 min of imaging. Following this, images were acquired every 5 min until the end of the imaging. Images were recorded at a higher frequency during the first 120 min to enhance the temporal resolution of images recording in-plane drying deformations within the SC. Imaging was conducted using a Nikon Eclipse Ti–U inverted microscope (Nikon, Melville, NY) equipped with a 1× objective lens (Nikon Plan UW), producing images at a resolution of 6.45 μm pixel^–1^. Images of samples were acquired with both brightfield and FITC filters at each time point. The design setup of the MEC system and microscope programming with respect to the imaging setup for capturing in-plane drying displacements is established, justified, and described in further detail in a prior study.^53,54^

### 4.18 Modeling Tissue Deformation

In-plane drying deformations of SC samples following a reduction to low R.H. conditions were analyzed by tracking the fluorescent bead displacement across time-lapsed images using particle image velocimetry (PIV).^69^ The in-plane radial displacements of the tracked fluorescent beads were azimuthally averaged and fitted to a previously designed model.^56,57,67^ The measured SC thickness (*h*_sc_ = 10 ± 3 μm), elastomer film elastic modulus (*E*_ef_ = 16 ± 1 kPa),^67^ and SC Poisson’s ratio (ν_SC_ = 0.4)^70^ were used to quantify dynamic changes in contractile drying stresses, *P_SC* and elastic moduli, *E_SC* of the drying SC samples. The changes in the contractile drying stress (*P_SC*) represent the internal force buildup due to water loss after the switch to low humidity conditions. Full details about the procedure for SC thickness measurements, model details of the radial displacement profiles, and quantification of both *P_SC* and *E_SC* are detailed in prior studies.^53,54^

### 4.19 Stimulated Raman Scattering (SRS) Microscopy

The stimulated Raman scattering (SRS) microscope was built using a dual-color, tunable near-infrared laser system (Insight X3, Spectra-Physics), which serves to generate the pump and Stokes beams for SRS imaging. To enhance the spectral resolution, a spectral focusing technique with high-dispersion glass rods was used. Amplitude modulation in the Stokes beam (fixed at 1045 nm) was achieved at a radiofrequency of 10 MHz using a resonant electro-optic modulator (EOM) (Thorlabs, Inc.). The pump and Stokes beams, after being temporally synchronized and collinearly combined, are focused onto the sample using a high numerical aperture (NA) water-immersion objective lens (NA = 1.1, CFI75-Apochromat-25XC-W-1300, Nikon Inc.). Following the interaction, the modulated Stokes beam is eliminated by a short-pass filter, while the pump beam, now carrying the stimulated Raman loss (SRL) signal, is detected by a large-area silicon photodiode (S3590–09, Hamamatsu Inc.). The signal from the photodiode is subsequently demodulated with a lock-in amplifier (HF2LI, Zurich Instruments) to extract the SRS signals. Laser-scanning imaging was performed by a two-dimensional linear galvanometer scanner (GVS002 and GPS011, Thorlabs Inc.), controlled by a data acquisition card (vDAQ, MBF Bioscience), ensuring accurate and efficient scanning for imaging. This laser-scanning SRS imaging system is controlled by the software ScanImage (MBF Bioscience).

### 4.20 Raman Confocal Spectroscopy

A Raman confocal spectroscopy system (Renishaw InVia) was used to measure the Raman spectra of Palmitoyl hexapeptide-12). The 785 nm laser line was used in this study. Aluminum foil was used as the substrate for the measurement.

### 4.21 *Ex Vivo* Skin Samples

Abdominoplasty *ex vivo* skin samples (Zen-Bio lot no. SKIN051319A; Ethnicity: Caucasian; Sex: Female; Age: 53 years; BMI: 27.5; Stretch Marks: Moderate) were received, washed twice in 1X PBS, treated with povidone-iodine solution (Ricca, Lot# 2611D26) to disinfect, washed again three times in 1X PBS and stabilized with 3X media −1X DMEM (Corning, Lot# 07719005), 10% Fetal Bovine Serum (Seradign, Lot# 182B15), 3% Penicillin/Streptomycin/Amphotericin B (Lonza, Lot# 18G025301) – overnight at 4 °C.

### 4.22 De-epithelialization and Decellularization

After stabilization, 12 mm biopsies were submerged in a de-epithelialization buffer overnight at 37 °C to remove the epidermis followed by a submersion in four changes of a decellularization buffer for 48 h at 37 °C using the protocol as stated in Kumar et al., 2013.^71^ De-epithelialization buffer consisted of 605 mg of Trizma (Sigma, lot no. 103 K5423), 4 g of NaCl (Sigma, lot no. 125H0988), and 202.5 mg of EDTA (Sigma, lot no. 30K0182) in 100 mL of 1X PBS. Decellularization buffer consisted of 1% Triton X-100 (VWR, lot no. 2126C348) and 0.25% Tributyl Phosphate (Sigma, lot no. MKCF5824) in 1X PBS. Following decellularization, the skins were washed 3 times in 1X PBS for 2 h each.

### 4.23 Treatment of Skins

The decellularized skins were submerged in PBS, 100 μg mL^–1^ Palmitoyl hexapeptide-12 for 5 days, with the solution changed daily. Three skins for each treatment were incubated at 37 °C.

### 4.24 Histochemistry

Skins were fixed overnight in 10% formalin, processed, paraffin-embedded, and microtome-sectioned (8 μm). Sections were then baked overnight at 50 °C and rehydrated before staining. An H&E stain was done to visualize if the skins were successfully decellularized and a Modified Verhoeff-Van Gieson stain (StatLab Item# KTVEL) was performed to visualize Elastin fibers according to the manufacturer’s protocol. Sections were mounted with Eukitt Quick-hardening mounting media (Sigma-Aldrich) and imaged using BX51 (Olympus) brightfield light microscopy at 20× magnification, white balanced, and exposure times of 57.87 and 11.39 ms for H&E and EVG, respectively. At least six images were taken per treatment.

### 4.25 Statistical Analysis

All experiments were carried out in at least triplicate (*n* = 3). Experimental data were presented as mean ± error, with error bars representing the standard error of the mean (SEM). Statistical significance of differences between means was performed using a two-way analysis of variance test (ANOVA) followed by Dunnett’s post hoc test for comparing treatments to PBS-submerged skins and Tukey’s multiple comparisons test for comparing the same treatment at different temperatures. Differences between mean values were considered significant for values of *p-value <0.05. Processing of data, graphs, and statistical analysis were all performed using GraphPad Prism v10.4.1.
